# Avoidant/Restrictive Food Disorder (ARFID), Food Neophobia, Other Eating-Related Behaviours and Feeding Practices among Children with Autism Spectrum Disorder and in Non-Clinical Sample: A Preliminary Study

**DOI:** 10.3390/ijerph20105822

**Published:** 2023-05-14

**Authors:** Agata Kozak, Kamila Czepczor-Bernat, Justyna Modrzejewska, Adriana Modrzejewska, Edyta Matusik, Paweł Matusik

**Affiliations:** 1 Non-public Health Care Center, Pediatric Neuropsychiatry Centre, 59-220 Legnica, Poland; 2 Department of Pediatrics, Pediatric Obesity and Metabolic Bone Diseases, Faculty of Medical Sciences in Katowice, Medical University of Silesia, 40-055 Katowice, Poland; 3 Institute of Pedagogy, University of Bielsko-Biała, 43-309 Bielsko-Biala, Poland; 4 Department of Psychology, Chair of Social Sciences and Humanities, School of Health Sciences in Katowice, Medical University of Silesia, 40-055 Katowice, Poland; 5 Department of Rehabilitation, Faculty of Health Sciences in Katowice, Medical University of Silesia, 40-055 Katowice, Poland

**Keywords:** avoidant/restrictive food disorder (ARFID), food neophobia, eating-related behaviours, feeding practices, children, autism spectrum disorder

## Abstract

Previous research shows that children with autism spectrum disorder (ASD) may experience several difficulties—including those related to eating—and this area of research needs to be explored further. This study had two main objectives: (1) comparison of the clinical (autism spectrum disorder) and non-clinical sample of children in terms of avoidant/restrictive food disorder, food neophobia, other eating-related behaviours and feeding practices; (2) assessment of selected predictors of food neophobia. The final sample included 54 children and parents from the clinical sample (ASD) and 51 from the non-clinical sample. Parents completed: the autism spectrum rating scales (ASRS), the eating disorders in youth questionnaire (EDY-Q), the children food neophobia scale (CFNS), the child eating behaviour questionnaire (CEBQ), the child feeding questionnaire (CFQ), and a socio-demographic survey. Our analysis allowed us to partially confirm the first hypothesis since the clinical sample (vs. the non-clinical group) had significantly higher scores in such variables as (a) avoidant/restrictive food disorder (ARFID), (b) food neophobia, (c) other eating-related behaviours: emotional under-eating, desire to drink, food fussiness, (d) feeding practices: pressure to eat. Moreover, our analysis of predictors of food neophobia in the clinical and non-clinical samples also allowed us to partially confirm the second hypothesis because it turned out that only in the clinical sample (vs. the non-clinical sample) were the predictors significantly associated with food neophobia, but only two of them (food fussiness and selective eating). In conclusion, our study showed that children with ASD (compared to children without this diagnosis) experience increased difficulties in eating behaviours, and their parents present a stronger intensity of the feeding practice based on pressure to eat. This study showed that feeding problems for children in the ASD sample are a significant problem, and it is still worth exploring this area in research.

## 1. Introduction

Nutrition is an important element for human functioning, and it plays a special role in children, as food provides them with necessary ingredients that allow them to grow and develop healthily [1]. In general, parents do not experience long-term difficulties in meeting the nutritional needs of their children, or their difficulties are temporary. However, sometimes it turns out that these difficulties are too serious to be solved on their own and parents have to seek help and support [1]. This situation can be encountered, among others, in case of children who receive a diagnosis of autism spectrum disorder (ASD). Children with this diagnosis require the appropriate support and therapy, and nutrition can have an impact conducted interventions [2]. In this manuscript, we will try to discuss the issues related to autism spectrum disorder, avoidant/restrictive food disorder (ARFID), food neophobia, other eating-related behaviours and feeding practices. The exploration of these topics is necessary, as there is an increasing number of children diagnosed with autism spectrum disorder, and some global statistics show that one in a hundred children is diagnosed with autism [3] and difficulties in feeding and eating among children with ASD were reported to occur in 50–90% of cases [4,5]. At the same time, there are reports in the literature indicating difficulties in the field of nutrition in this clinical sample (e.g., related to rigid and selective food consumption; [6]) and this topic is discussed, among others, in relation to ARFID [2,7]. Therefore, it was decided to analyse the issue of autism spectrum disorder while referring to the assessment of various aspects related to the way of how this group of children eats and is fed. This decision was also made due to the fact that (although we already have some interesting findings in this area; e.g., [6,7,8]) this area requires further exploration, and knowledge about some of the analysed aspects is still being systematised (e.g., ARFID was included in the ICD-11 as a new diagnosis; [9]).

In general, autism spectrum disorder is defined as persistent deficits in communication and social interaction, and rigid, repetitive, and restricted patterns of behaviour, activities, and interests that are markedly excessive or nontypical given the person’s age and surrounding sociocultural context [10,11]. Symptoms of this disorder tend to manifest usually during early childhood and may become fully apparent when social demands exceed the child’s capabilities. The deficits described above manifest themselves in various life situations (although their intensity may vary depending on the context). However, the severity of these conditions is so high that they cause significant impairment in various spheres of life [10,11]. The broad literature contains numerous detailed descriptions of the difficulties experienced in autism spectrum disorder diagnosis [12,13,14,15]. Delecato [12] described that people with autism spectrum disorder often report difficulties with their senses of hearing, taste, smell, sight, touch, balance, and kinaesthetic sense [12]. They complain about too high or too low sensitivity to stimuli. This might be the reason why these people take various actions to regulate the reception of unpleasant stimuli. Delecato [12] believes that, in some cases, children with autism may experience severe difficulties in the sense of taste and smell and, therefore, may have various eating problems. Very sensitive children are reluctant to accept food because the taste is too intense for them. Less sensitive children can eat anything, including inedible and dangerous things because they are looking for intense taste sensations [12]. In turn, Bluestone [13] described her own eating problems in relation to autism. She highlighted numerous sensory difficulties. The author was very sensitive to sounds and struggled with chewing problems. Specifically, she did not like the sensation of chewing her food. She also had a problem with muscle tone, and, thus, with assessing how hard she should clench her jaw on a certain type of food. The “noise” in the mouth during chewing was very unpleasant for her. She recalls that the noises accompanying her eating were unbearable for her and caused great fear. This may mean that the difficulties that will arise in the field of nutrition in patients with autism spectrum may also be related to the consistency of food and the fact that people with the disorder are unable to accept and chew a given food. At the same time, it should be noted that the above-described feeding difficulties among children with ASD are as relating to hyper-or hyposensitivity to various properties of foods or liquids (e.g., flavour, temperature, texture). This is a plausible explanation, especially given the vocal reports of some patients with ASD. However, there is no evidence to show, explicitly or definitively, that feeding difficulties among children with ASD are due to sensory deficits or excesses. It could be that some children display an intense preference for some foods, and as a result, have a severely limited diet variety due to their insistence on sameness or restrictive interests [10,11,12,13,14,15]. Additionally, in the context of feeding difficulties, resistance to change or insistence on sameness is a core feature of ASD and often leads to extreme emotional outbursts in response to even small changes in the environment [10,11,12,13,14,15,16]. Referring to both ASD and ARFID, a method to confirm a consistent etiology of feeding disorders was not identified, and it was often found that it varies dramatically. It should be remembered that some children with ASD and ARFID also have complex medical histories (e.g., history of gastroesophageal reflux disease, food allergies) which could also serve as a contributing factor to the development and maintenance of these disorders [6,8,16,17,18,19]. Other children with ARFID who relied on lower textures or textures that melt easily in the mouth with minimal bite pressure (e.g., meltable solid foods such as crackers or cheese puffs) may acquire oral-motor deficits. With limited opportunities to refine chewing skills, children may find that management of table-textured food is too challenging or a great amount of response effort; therefore, they continue to rely on lower textures for the majority of their intake [6,8,16,17,18,19]. Importantly, not chewing food leads to numerous digestive problems, such as constipation and diarrheal which may cause aversions to a given group of products in children [13]. In addition, Delecato [12] noted that many children on the spectrum struggle with food intolerances, which makes it even more difficult for parents to choose the right type of food. These are some of the primary difficulties and challenges with diagnostic nosology and feeding practises among children with ASD and ARFID. All these factors can contribute to numerous problems in terms of proper nutrition and feeding of children [12]. Moreover, there is a pattern of food choices in children with ASD [15]. Namely, these children are reluctant to eat fruit and vegetables (which contributes to micronutrient deficiencies) and are more likely to choose carbohydrate and high-fat products which may increase the risk of being overweight and obese [15].

As noted above, many ASD children also have eating and feeding problems [20,21]. One meta-analysis showed that children with ASD are up to 5 times more likely to experience these difficulties than those without ASD [22], and children with ASD and ARFID have more problems with eating (avoiding food) and are more sensory sensitive than children from the non-clinical sample [23]. With regard to these findings, it turns out that ASD autism co-occurs with avoidant/restrictive food disorder (ARFID) in up to 12.5–33.3% patients [24,25,26]. Avoidant-restrictive food intake disorder (ARFID) is related to restriction/avoidance of food intake that may have the following consequences (both or one of them; [27,28]): (1) difficulties in the field of nutrition related to the qualitative and quantitative diet balance negatively affecting physical health (e.g., resulting in significant weight loss and nutritional deficiencies, dependency on oral nutritional supplements or tube feeding); (2) significant impairment in various spheres of life resulting, among others, from severe stress associated with the need to avoid social situations related to food consumption. Avoidance/restriction is not related to a desire to lose weight/change body shape and be overly concerned with the body. These eating patterns and their consequences are also not caused by lack of access to food or other physical or mental diseases and their treatment. What is equally relevant [27,28,29]: (1) depending on the patient’s experiences, many reasons for avoidance/restriction can be distinguished (e.g., sensory characteristics of food, lack of general interest in food, aversive experiences in which the child, for example, vomited after consuming some food or choked on it), but, at the same time, it should be remembered that that there may also be a situation in which it will not be possible to identify the triggers of this disorder; (2) patients may be characterized by a lack of interest in eating, feeling low appetite or having difficulties in capturing and distinguishing hunger and satiety signals, as well as the fact that their eating patterns may be dependent on psychoemotional factors (e.g., eating under emotional arousal, refusing food as a manifestation of resistance, a high level of distractibility) and may require additional feeding practises (to encourage children to eating) from parents and others in the immediate environment; (3) ARFID generally does not result in difficulties in consuming foods that a person prefers (hence, it may not always be accompanied by being underweight); (4) ARFID may negatively affect family functioning related to feeding and eating (eating meals together may be associated with increased stress, there may be greater irritability during feeding, children may try hard to force what and how much they will eat). It should also be mentioned here that both ASD and ARFID individuals may have significant eating difficulties linked to food sensory characteristics. However, people with ASD encounter other difficulties described in the paragraph above, and difficulties in nutrition may also result from a strong attachment to strictly defined procedures (which may possibly be related to, among others, preparing a meal; [27] ICD-11). At the same time, it is emphasized that if restricted or avoided eating in a person with ASD resulted in significant weight loss or health consequences or functional impairment, ARFID may be assigned as well [27].

Interestingly, other research showed that there are links between autism spectrum disorders and food neophobia (manifested by difficulties in the child’s acceptance of new foods and a general reluctance to try them, and in non-clinical samples recognized as a normal stage in child development often between the ages of 2 and 6; [15,30,31]). In one study, children with ASD were rated as more neophobic than their peers in the control group [15]. Furthermore, it was found that high levels of autistic traits may increase the risk of being underweight [15]. Analysing the literature on food neophobia, it was found that that parents’ eating practices may be significant here. This is because the more neophobic attitudes of mothers are, the higher their children’s neophobia level [32]. In this context, it should also be mentioned that in many situations where parents experience difficulties in feeding their children, they will use “strategies” that they feel are intended to solve the existing problem [33]. Some of these practices may be particularly ineffective and reinforce maladaptive behaviours in children. An example of a behaviour that has a negative impact during feeding is distracting the child by watching cartoons or fetching toys. Other examples may be the lack of autonomy of children in terms of the choice of food, the amount of their consumption and the act of eating, as well as exerting pressure to eat the number of meals that the parents set (without considering the level of hunger and satiety of the child), or the use of food as a reward or emotional state regulator [33,34,35,36]. With reference to these outcomes, other studies looked for a relationship between the eating behaviours of children with ASD and children without this diagnosis, and their parents’ feeding practises [37]. This study showed that parental rewards reinforce refusal to eat and reduce meal variety in a non-clinical sample. In addition, in the group of children with ASD, the use of such practices was negatively correlated with the children’s eating pleasure and these children were more likely to refuse food, and their parents paid more attention to preparing special meals for them [37].

To sum up, previous research showed that children with autism spectrum disorder may experience several difficulties—including those related to eating—and this area should be further explored. This topic is extremely important because an incorrect diet may result in deficiencies of certain nutrients, and this disorder accompanies a person throughout his life. Appropriate therapy (including activities related to balanced diet support), and parental support for healthy feeding practices can help children in later functioning. Behaviour therapy and functional analysis, the methods of which were analysed in many studies, provide a lot of important guidelines in the field of feeding and eating management (e.g., [8,18,19,38,39]). What is important, knowledge in the field of eating behaviour and feeding of children with ASD and ARFID is developing dynamically. Therefore, this study had two main objectives: (1) comparison of the clinical (autism spectrum disorder) and non-clinical sample of children in terms of: avoidant/restrictive food disorder, food neophobia, other eating-related behaviours (this category includes the following variables that are associated with such feeding and/or eating difficulties as: (a) satiety responsiveness—tendency to the adequate appearance of the feeling of satiety (e.g., *My child cannot eat a meal if s/he has had a snack just before*), (b) slowness in eating—tendency to eat meals slowly (e.g., *My child eats slowly*), (c) food fussiness—tendency to be negative about certain products and to eat new and/or different foods (e.g., *My child is difficult to please with meals*), (d) food responsiveness—the tendency of the child to eat without considering satiety (e.g., *Given the choice, my child would eat most of the time*), (e) enjoyment of food—the tendency to become preoccupied with food and to be guided by pleasure in food choices (e.g., *My child enjoys eating*), (f) desire to drink—a tendency to drink a lot (e.g., *If given the chance, my child would always be having a drink*), (g) emotional undereating—tendency to reduce food intake under the influence of emotions (e.g., *My child eats less when angry*), (h) emotional overeating—tendency to increase food intake under the influence of emotions (e.g., *My child eats more when worried*) and feeding practices (perceived responsibility, restriction, parents’ concerns about child weight, pressure to eat, monitoring); (2) assessment of selected predictors (sensory sensitivity, food fussiness, enjoyment of food, feeding practise (concern about child weight, pressure to eat, monitoring), selective eating) of food neophobia. Based on previous work, it was hypothesised (hypothesis 1) that: (1a) a clinical sample (compared to a non-clinical one) would have a higher level of ARFID-related features and (1b) a higher level of food neophobia, and (1c) would have less adaptive other eating-related behaviours; (1d) parents of a clinical sample (compared to a non-clinical one) would exhibit less adaptive feeding practices. Moreover, it was assumed (hypothesis 2) that the selected predictors listed above would be significantly and positively associated with food neophobia, but the model of the relationship between these variables would be significant only in the clinical sample.

## 2. Materials and Methods

### 2.1. Participants and Procedure

Our preliminary study was conducted from October 2021 to January 2022. All procedures were conducted in accordance with the Declaration of Helsinki and the study was approved by the Ethics Committee. At the beginning of the study, all participants were informed about the aim of the study, anonymity, and voluntariness (with information about the possibility of withdrawing without giving a reason) followed by consent to participate in the study and if they expressed their willingness to participate, they were given a set of measures to complete. The children’s parents provided information on their functioning. Children and parents were not remunerated for their participation.

Children aged 3 to 15 were divided into two groups: (a) clinical sample (autism spectrum disorder (ASD)—diagnosis of autism or Asperger’s syndrome): *N* = 54, (b) non-clinical sample (without a diagnosis of autism spectrum disorder and other disorders related to the development and mental health): *N* = 51. The clinical group was recruited in the specialized centre dealing with the support of children with autism spectrum disorder (Pediatric Neuropsychiatry Centre) and the diagnosis was made by a team consisting of a psychiatrist, psychologist, and neurologist (based on testing, e.g., the Autism Spectrum Rating Scales [40]) and observation). Parents of children with ASD completed the paper version of the measures. After receiving the questionnaires, parents were asked to complete them on their own, seal them in a previously prepared envelope, and return them to the researcher. Parents of the non-clinical sample (children without an ASD diagnosis) filled in the online version of the measures. They were volunteers from the various parents’ groups on Facebook. They were sent a link to a Google Form and asked to complete the questionnaires. Parents did not receive additional guidance on completing questionnaires and interpreting the items.

Detailed characteristics of the children and their parents are presented in Table 1.

### 2.2. Measures

#### 2.2.1. The Autism Spectrum Rating Scales (ASRS)

Parents completed the 70/71-item (2–5/6–18 years of age) ASRS, rating each item on a 5-point scale ranging from 0 (*never*) to 5 (*very frequently*) [40,41]. This scale assesses the symptoms of autism spectrum disorders (ASD). It consists of six general clinical scales and two or three (version for 2–5 or 6–18 years of age) additional ASRS scales (social/communication, unusual behaviours, self-regulation, peer socialization, adult socialization, social/emotional reciprocity, atypical language, stereotypes, behavioural rigidity, sensory sensitivity, attention). The higher the score, the more severe the symptoms of autism spectrum disorders. As already mentioned above, ASRS was also used by a psychiatrist, psychologist, and neurologist in the diagnosis of autism spectrum disorder. In addition, the sensory sensitivity scale (e.g., *Overreacting to common smells*) was used in our regression analyses. Reliability and validity were acceptable [40,41] and Cronbach’s alpha coefficient for the sensory sensitivity scale was 0.74 (2–5 years of age) and 0.88 (6–18 years of age).

#### 2.2.2. The Eating Disorders in Youth-Questionnaire (EDY-Q)

Parents were also asked to complete the eating disorders in youth questionnaire [42]. This questionnaire consists of 14 items that measure assessing symptoms of avoidant/restrictive food disorder (ARFID—12 items involving three subscales: food avoidance emotional disorder (FAED), selective eating (SE), and functional dysphagia (FD)) and pica and rumination disorder). Items were rated on a 7-point scale ranging from 1 (*never true*) to 7 (*always true*). In this study, only 12 items were used to describe ARFID and separately a subscale of selective eating (e.g., *My child does not like to try food with a specific smell, taste, appearance, or a certain consistency (e.g., crispy, or soft*). The higher the scores, the more severe the ARFID symptomatology. Importantly, in the original version, this method was self-reported, but due to the age and disorders of the clinical sample, the authors could not use the method in this version and a version was created in which parents assessed their children’s functioning. Reliability and validity were acceptable [42] and Cronbach’s alpha coefficient for the general scale of AFRID was 0.81, and for the selective eating, subscale was 0.92.

#### 2.2.3. The Children Food Neophobia Scale (CFNS)

To measure food neophobia, the 6-item CFNS scale was used [31,43] with items rated on a 4-point scale (from 1—*strongly disagree* to 4—*strongly agree*, e.g., *My child does not trust new food*). The higher the score, the greater the food neophobia. Reliability and validity were acceptable [31,43] and Cronbach’s alpha coefficient was 0.92.

#### 2.2.4. The Child Eating Behaviour Questionnaire (CEBQ)

Parents completed also the 35-item child eating behaviour questionnaire, which measures parents’ assessment of children’s eating-related behaviours [44,45]. It consists of eight subscales (food responsiveness, emotional over-eating, enjoyment of food, desire to drink, satiety responsiveness, slowness in eating, emotional under-eating, food fussiness, e.g., *my child is difficult to please with meals, my child eats more when annoyed, if allowed to, my child would eat too much*). Responses are rated on a 5-point scale (from 1—*never* to 5—*always*). The scores of all subscales (except for satiety responsiveness, slowness in eating) are interpreted so that higher scores indicate less adaptive eating-related behaviours. All subscales were used in this study. Reliability and validity were acceptable [44,45] and the Cronbach’s alpha coefficient was: (a) food responsiveness: 0.86, (b) emotional under-eating: 0.68, (c) enjoyment of food: 0.84, (d) satiety responsiveness: 0.65, (e) desire to drink: 0.80, (f) slowness in eating: 0.79, (g) emotional over-eating: 0.82, (h) food fussiness: 0.82.

#### 2.2.5. The Child Feeding Questionnaire (CFQ)

To measure parents’ feeding practices, the 31-item CFQ was used [46] which consists of seven subscales: restriction, pressure to eat, monitoring, the perception of responsibility, parent perceived weight, perceived child weight, parents’ concerns about child weight. Items were rated on a 5-point scale (changing depending on the subscale/question). In this study, we used almost all subscales (two subscales—parent perceived weight, and perceived child weight—were not used due to serious data gaps). Higher scores, therefore, mean a feeding style characterized by greater: (a) restriction (e.g., *I offer sweets (candy, ice cream, cake, pastries) to my child as a reward for good behavior*), (b) pressure to eat (e.g., *My child should always eat all of the food on her plate*), (c) monitoring (e.g., *How much do you keep track of the sweets (candy, ice cream cake, pies, pastries) that your child eats?*), (d) perceived responsibility (e.g., *How often are you responsible for deciding what your child’s portion sizes are?*), (e) parents’ concerns about child weight (e.g., *How concerned are you about your child having to diet to maintain a desirable weight?*). Reliability and validity were acceptable [46] and the Cronbach’s alpha coefficient was: (a) sense of responsibility: 0.60, (b) restriction: 0.75, (c) parents’ concerns about child weight: 0.86, (d) pressure to eat: 0.81, (e) monitoring: 0.87.

#### 2.2.6. Socio-Demographic Survey and Additional Questions

Parents completed questions about: (a) the parent’s and child’s gender, age, weight, height, (b) the parent’s education level, (c) the financial status of the family, (d) the parent’s marital status, (e) the number of children in the family, (f) the diagnosis of development and mental health disorders (including autism spectrum disorder).

### 2.3. Statistical Analysis

Statistica™ 12 [47] software (Tibico Sotware Inc., Palo Alto, CA, USA) was used to carry out Student’s *t*-test for independent variables and linear regression analysis. All assumptions necessary for Student’s *t*-test were met (continuous data, homogeneity of variance, normal distribution, equal samples). It was used to compare the clinical (autism spectrum disorder) and non-clinical sample of children in terms of avoidant/restrictive food disorder (ARFID), food neophobia, other eating-related behaviours (responsiveness, slowness in eating, food fussiness, food responsiveness, enjoyment of food, desire to drink, emotional undereating, emotional overeating) and feeding practices (perceived responsibility, restriction, parents’ concerns about child weight, pressure to eat, monitoring). The linear regression model (variable selection methods: enter) was applied to assess selected predictors of food neophobia (dependent variable) separately in the clinical and non-clinical samples (the tested predictors: sensory sensitivity, food fussiness, enjoyment of food, feeding practice (i.e., concern about child weight, pressure to eat, monitoring), selective eating).

## 3. Results

### 3.1. Comparison Analyses

In order to verify the first hypothesis, comparisons were made using Student’s *t*-test (Table 2).

To sum up, the analysis using Student’s *t*-test showed that the clinical sample (compared to the non-clinical): (1a) had a significantly higher level of intensity of ARFID symptoms, (1b) had a significantly higher level of food neophobia, (1c) had a significantly higher level in relation to other eating-related behaviours such as: higher levels of such aspects of eating behaviours as emotional under-eating, desire to drink and food fussiness, and did not differ in terms of the other CEBQ subscales. Moreover, parents of children with ASD presented a higher level of eating pressure than in the group without this diagnosis and parents did not differ in other feeding practices (1d).

### 3.2. Regression Analyses

To verify the second hypothesis, the relationships between food neophobia and its predictors were analysed separately in the clinical and non-clinical samples (Table 3 and Table 4; see the supplementary material Table S1 for additional information on relationships between variables).

To sum up, the regression analyses showed that: (a) in the clinical sample, the proposed model turned out to be a good fit to the data, and food fussiness and selective eating were the significant predictors of food neophobia (both variables associated with a positive relationship with food neophobia), (b) in the non-clinical sample, the proposed model was not found to fit the data well and none of the predictors were significant.

## 4. Discussion

The main objective of the study was to: (1) compare the clinical (autism spectrum disorder; ASD) and non-clinical sample of children in terms of: avoidant/restrictive food disorder (ARFID), food neophobia, other eating-related behaviours (responsiveness, slowness in eating, food fussiness, food responsiveness, enjoyment of food, desire to drink, emotional undereating, emotional overeating), and feeding practices (perceived responsibility, restriction, parents’ concerns about child weight, pressure to eat, monitoring); (2) assess selected predictors (sensory sensitivity, food fussiness, enjoyment of food, feeding practise (concern about child weight, pressure to eat, monitoring), selective eating) of food neophobia separately in the clinical and non-clinical sample.

Our analysis allowed us to partially confirm the first hypothesis since the clinical sample (autism spectrum disorder sample vs. the non-clinical group) had significantly higher scores in such variables as: (a) avoidant/restrictive food disorder (ARFID), (b) food neophobia, (c) other eating-related behaviours: emotional under-eating, desire to drink, food fussiness, (d) feeding practices: pressure to eat. In terms of other variables (other CEBQ and CFN subscales), the compared groups did not differ.

With regard to ARFID, our outcomes are consistent with previous studies [48,49] which analysed data from over 2000 children (including 1462 children with autism) and showed that atypical eating behaviours were significantly more frequent in the group of children with autism (70.4%) than in children with other disorders (including ADHD and intellectual disability) (13.1%) or in typically developing children (4.8%). Additionally, various authors showed that there is a significant relationship between ARFID and ASD [22,24,25,26,48,49]. Similar conclusions can be drawn regarding food neophobia. This is because our results are also consistent with previous research that indicated a significant relationship between food neophobia and ASD [15].

As mentioned above, in the field of other eating-related behaviours and feeding practices, significant differences were also noticed between the clinical and non-clinical samples. With regard to the first of these areas, it turned out that children from the clinical sample (compared to the non-clinical sample): (a) initially refused to try new foods more often or disliked trying them more, (b) consumed a smaller variety of foods, (c) showed less interest in new food, (d) more often stated that they did not like the food even without trying it, (e) more often asked for something to drink at the meal and drank a lot during the day, (f) ate less when they were angry, tired or sad, and even more so when they were happy. Importantly, partially similar results were obtained by researchers Öz and Bayhan [37]. In the cited studies, children with ASD also had significantly higher levels of fastidiousness than children without this diagnosis; however, no significant differences were found in the desire to drink and emotional undereating [37]. In relation to feeding practices, parents of children with ASD (compared to the non-clinical sample) exerted stronger pressure for their children to eat whole meals prepared by them, were more vigilant about whether their child was eating enough, and more strongly urged their children to eat when they declared that they are not hungry. Other researchers also observed that parents of children declaring a small appetite put more pressure on food consumption than parents of children declaring a subjectively higher appetite [34]. In both studies, therefore, a similar mechanism was observed in which parents of children who eat were reluctant to put more pressure on them to eat (in their opinion) enough. However, it must be stressed that, referring to our findings, the higher pressure to eat could be because parents had more feeding problems [7,18,19,38,39]. Therefore, the mechanism of exerting pressure to eat should be further analysed in terms of its effectiveness and short-term and long-term effects, to create treatment implications and practical guidelines for parents [6,7,8,16,17,38,39].

Our analysis of predictors of food neophobia in the clinical and non-clinical samples also allowed us to partially confirm the second hypothesis because it was found that only in the clinical sample (autism spectrum disorder sample vs. the non-clinical sample) were the predictors significantly associated with food neophobia, but only two of them (food fussiness and selective eating). The other predictors were not significantly associated with the dependent variable in the clinical sample. Referring to the obtained outcomes, the selection of predictors was guided by findings from numerous works [12,13,37,50,51,52] in which the importance of selected variables in the context of the functioning of people with ASD was repeatedly emphasized. It turned out that only in the group of children with ASD could we predict the level of food neophobic tendency based on persistent, incorrect behaviours and eating attitudes, and these predictors are food fussiness and selective eating which may indicate that selectiveness, based on various sources (e.g., food’s sensory characteristics, strictly defined procedures related to preparing a meal; oral motor difficulties associated with the consumption of various foods), may contribute to persistent reluctance to try new products which do not disappear with the achievement of an appropriate developmental age [52]. However, this is only a preliminary study and further analyses in this regard are necessary, since the study was not free from the limitations mentioned below.

## 5. Conclusions

In conclusion, our study showed that children with ASD (compared to children without this diagnosis) show greater difficulties in the field of some eating behaviours, and their parents present a more vigorous intensity of the feeding practice based on pressure to eat. These studies showed that problems with feeding children in the ASD sample are a significant problem and it is still worth exploring this area in research. This was also confirmed by other studies which showed that atypical eating behaviours are 15 times more common in children with autism than in healthy children [48,49]. This explains why it is of great importance for specialists to explore the knowledge of autism spectrum disorder and eating disorders [48,49]. Importantly, Mayes and Zickgref [48,49] mentioned that the atypical eating behaviours seen in autism occur at a very young age and affect about 70% of all people with ASD. Therefore, primary care and family physicians should be sensitive to such issues and refer children for an autism spectrum diagnosis [48,49]. Interestingly, some authors indicated that putting pressure on children during a meal is not an appropriate method of dealing with children’s fussiness and parents often lack other methods to help them cope with a difficult situation [53,54,55]. However, these are reports based mainly on samples from the general population. In the field of support for children with ASD, many studies referred to the use of functional analysis methods as an effective method of assistance (e.g., [56,57]). It is worth remembering that parents of children with ASD may often choose a strategy based on pressure to eat because they are concerned about the child with ASD becoming underweight or having other serious physical health consequences. Moreover, in the general population such an attitude may favour the emergence of significant resistance to eating (which may manifest itself in the form of fussiness or high selectivity in eating) [53,54,55]. This may also result in difficulties in interpreting the physiological signals of hunger, satiety, and thirst, as well as the inability to distinguish emotions from hunger and satiety signals. Strong pressure can also become a source of aversive experiences which may appear especially often when consuming new and unfamiliar products by the child [53,54,55]. Thus, the context of feeding behaviour in the clinical and non-clinical population can be completely different. Therefore, it is worth conducting further research among children with ASD and their parents to take a closer look at the strategy based on exerting pressure and analyse its impact on the child’s physical and mental health (including his relationship with food, food aversion). Parents are the first educators of their children; therefore, the level of their knowledge about healthy eating affects not only their own eating habits, but also the eating habits of their children. Therefore, it is important to educate parents in the field of choosing a properly balanced diet for their child and proper behaviours related to eating and feeding. Furthermore, future studies should attempt to answer the question of what parents’ feeding practises are conducive to maintaining a healthy and balanced diet in children with a high level of fastidiousness in terms of nutrition. It is also important to conduct further research looking for more sufficient methods of treating nutritional abnormalities amongst patients with ASD (especially in the group with accompanying ARFID and obesity).

Finally, it is worth emphasizing that these studies had several limitations that should be eliminated by conducting further analyses in this area: (a) this was a cross-sectional study in which a relatively small sample took part (especially when referring to its size for regression analysis), recruited using voluntary sampling design, (b) the study was conducted in paper form for the clinical sample and in online form (online survey) for the non-clinical sample (which was caused by restrictions related to the SARS-CoV-2 pandemic), (c) the measurement was based only on subjective reports from caregivers from a small sample size, (d) not all measures were validated in the Polish sample (publications on the validation of some measures appeared only after the beginning of our study, e.g., [58,59]), (e) the original version of the EDY-Q is a self-report measure (in this study, it was used in the version in which parents assessed the functioning of their children), (f) the reliability of some CEBQ and CFQ subscales could be improved, (g) sensory sensitivity was assessed using ASD diagnosis measure (ASRS); therefore, it was not a tool dedicated to an in-depth diagnosis of sensory sensitivity in the clinical and non-clinical sample. Finally, it should be emphasized that since there could be masked elements contributing to the feeding challenges among these children (e.g., resistance to change, hypo- or hyper sensitivities, oral-motor skill deficits, medical complexity, cognitive and adaptive functioning abilities, genetic disorders), further research needs to analyse the background of these difficulties and challenges much more carefully.

## Figures and Tables

**Table 1:** Characteristics of parents and children by clinical and non-clinical sample.

	Clinical Sample *N* = 54	Non-Clinical Sample *N* = 51
	*M* (*SD*)	
**Children’s age**	7.44 (3.06)	6.71 (3.22)
**Parents’ age**	37.28 (6.05)	34.90 (5.06)
**Children’s body mass index (BMI) (kg/m^2^)**	17.08 (4.08)	16.74 (3.32)
**Parents’ body mass index (BMI) (kg/m^2^)**	26.31 (4.87)	24.32 (4.06)
	*N* (%)	*N* (%)
**Education level**		
Elementary	1 (1.85%)	0
Lower secondary	3 (5.56%)	0
Vocational	13 (24.07%)	2 (3.92%)
Secondary	22 (40.74%)	14 (27.45%)
Higher	15 (27.78%)	35 (68.63%)
**Financial status**		
Definitely good	5 (9.26%)	11 (21.57%)
Rather good	49 (90.74%)	38 (74.51%)
Rather bad	0	2 (3.92%)
Definitely bad	0	0
**Marital status**		
Single	8 (14.82%)	6 (11.77%)
Cohabitation	6 (11.11%)	3 (5.88%)
Married	32 (59.26%)	37 (72.55%)
Divorced	7 (12.96%)	5 (9.80%)
Widowed	1 (1.85%)	0
**Number of children in the family**		
1	19 (35.19%)	13 (25.49%)
2	24 (44.44%)	29 (56.86%)
3	8 (14.82%)	9 (17.65%)
4	2 (3.70%)	0
5	1 (1.85%)	0
**Diagnosis**		
autism	48 (88.89%)	0
Asperger’s syndrome	6 (11.11%)	0

**Table 2:** Comparison of the clinical and non-clinical samples.

	Clinical Sample	Non−Clinical Sample			
	*M* (*SD*) [95% CI ^1^]	*M* (*SD*) [95% CI ^1^]	Student’s *t*−Test	*p*	Cohen’s *d*
Hypothesis 1a—avoidant/restrictive food disorder (EDY−Q)					
**ARFID**	**34.77 (12.93)** **[31.32–38.22]**	**26.61 (9.05)** **[24.13–29.09]**	***t*** **(103) = 3.73**	**<0.001**	**0.73**
Hypothesis 1b—food neophobia (CFNS)					
**Food neophobia**	**17.57 (4.29)** **[16.43–18.71]**	**14.18 (3.64)** **[13.18–15.18]**	***t*** **(101) = 4.30**	**<0.001**	**0.85**
Hypothesis 1c—other eating-related behaviours (CEBQ)					
Food responsiveness	11.91 (4.67) [10.66–13.16]	11.96 (4.62) [10.69–13.23]	t(103) = −0.06	>0.05	0.01
**Emotional under−eating**	**10.87 (2.85)** **[10.11–11.63]**	**9.55 (3.27)** **[8.65–10.45]**	***t*** **(103) = 2.21**	**<0.05**	**0.43**
Enjoyment of food	12.65 (3.48) [11.72–13.58]	13.57 (3.20) [12.69–14.45]	*t*(103) = −1.41	>0.05	0.28
Satiety responsiveness	14.67 (3.07) [13.85–15.49]	13.82 (3.16) [12.95–14.69]	*t*(103) = 1.38	>0.05	0.27
**Desire to drink**	**10.07 (2.91)** **[9.29–10.85]**	**8.73 (2.61)** **[8.01–9.45]**	***t*** **(103) = 2.49**	**<0.05**	**0.49**
Slowness in eating	12.11 (3.80) [11.10–13.12]	11.18 (2.78) [10.42–11.94]	*t*(103) = 1.43	>0.05	0.30
Emotional over−eating	7.60 (3.06) [6.78–8.42]	7.90 (3.95) [6.82–8.98]	*t*(102) = −0.43	>0.05	0.08
**Food fussiness**	**21.74 (5.02)** **[20.40–23.08]**	**17.75 (5.30)** **[16.30–19.20]**	***t*** **(102) = 3.94**	**<0.001**	**0.77**
Hypothesis 1d—feeding practices (CFQ)					
**Pressure to eat**	**10.20 (4.82)** **[8.91–11.49]**	**7.54 (3.24)** **[6.65–8.43]**	***t*** **(103) = 3.29**	**<0.001**	**0.65**
Perceived responsibility	12.72 (1.84) [12.23–13.21]	12.20 (2.23) [11.59–12.81]	*t*(103) = 1.32	>0.05	0.25
Parents’ concerns about child weight	5.26 (2.98) [4.47–6.05]	4.78 (2.89) [3.99–5.57]	*t*(103) = 0.83	>0.05	0.16
Restriction	26.98 (1.68) [26.53–27.43]	24.57 (1.73) [24.10–25.04]	*t*(103) = 1.76	>0.05	1.41
Monitoring	11.37 (2.47) [10.71–12.03]	11.88 (2.55) [11.18–12.58]	*t*(103) = −1.04	>0.05	0.20

Significant differences are marked in bold; EDY-Q—the Eating Disorders in Youth-Questionnaire; CFNS—the Children Food Neophobia Scale; CEBQ—the Child Eating Behaviour Questionnaire; CFQ—the Child Feeding Questionnaire; ^1^ The confidence intervals given are for *M*.

**Table 3:** Linear regression model for the prediction of food neophobia in the clinical sample.

	Dependent Variable: Food Neophobia (CFNS)				
	*F*(7, 45) = 17.74; *p* < 0.001; *R*^2^*_adj._* = 0.69				
Predictors:	B	SE	*β*	*t*	*p*
Sensory sensitivity (ASRS)	−0.08	0.09	−0.06	−0.92	>0.05
**Food fussiness** (CEBQ)	**0.53**	**0.16**	**0.45**	**3.41**	**<0.001**
Enjoyment of food (CEBQ)	0.12	0.10	0.15	1.23	>0.05
Concern about child weight (CFQ)	−0.14	0.08	−0.20	−1.76	>0.05
Pressure to eat (CFQ)	0.08	0.09	0.07	0.88	>0.05
Monitoring (CFQ)	0.12	0.09	0.19	1.28	>0.05
**Selective eating** (EDY-Q)	**0.35**	**0.15**	**0.25**	**2.27**	**<0.05**

Significant predictors are marked in bold; CFNS—the Children Food Neophobia Scale; ASRS—the Autism Spectrum Rating Scales; CEBQ—the Child Eating Behaviour Questionnaire; CFQ—the Child Feeding Questionnaire; EDY-Q—the Eating Disorders in Youth-Questionnaire.

**Table 4:** Linear regression model for the prediction of food neophobia in the non-clinical sample.

	Dependent Variable: Food Neophobia (CFNS)				
	*F*(7, 41) = 0.60; *p* > 0.05; *R*^2^*_adj._* = −0.06				
Predictors:	B	SE	*β*	*t*	*p*
Sensory sensitivity (ASRS)	0.05	0.19	0.04	0.24	>0.05
Food fussiness (CEBQ)	0.03	0.29	0.02	0.09	>0.05
Enjoyment of food (CEBQ)	0.11	0.26	0.13	0.43	>0.05
Concern about child weight (CFQ)	−0.31	0.21	−0.39	−1.50	>0.05
Pressure to eat (CFQ)	−0.16	0.18	−0.18	−0.90	>0.05
Monitoring (CFQ)	−0.06	0.19	−0.09	−0.33	>0.05
Selective eating (EDY-Q)	0.07	0.24	0.05	0.30	>0.05

Significant predictors are marked in bold; CFNS—the Children Food Neophobia Scale; ASRS—the Autism Spectrum Rating Scales; CEBQ—the Child Eating Behaviour Questionnaire; CFQ—the Child Feeding Questionnaire; EDY-Q—the Eating Disorders in Youth-Questionnaire.

## Data Availability

The data that support the findings of this study are available from the corresponding author upon reasonable request.

## Supplementary Materials

The following supporting information can be downloaded at: https://www.mdpi.com/article/10.3390/ijerph20105822/s1, Table S1: Our results of correlation analysis.
