# Supplementary material for: Avoidant/Restrictive Food Disorder (ARFID), Food Neophobia, Other Eating-Related Behaviours and Feeding Practices among Children with Autism Spectrum Disorder and in Non-Clinical Sample: A Preliminary Study
Source: Int J Environ Res Public Health. 2023 May 14;20(10):5822. doi: 10.3390/ijerph20105822 (PMC10218647; doi:10.3390/ijerph20105822)
Supplement: Supplementary file 1 [file ijerph-20-05822-s001.zip › ijerph-2254505-SI.pdf]

Table S1: Our results of correlation analysis.

Clinical sample

N = 54

Zmienna	Sensory sensitivity (ASRS)	Food fussiness (CEBQ)	Enjoyment of food (CEBQ)	Concern about child weight (CFQ)	Pressure to eat (CFQ)	Monitoring (CFQ)	**Selective eating** (EDY-Q)
Dependent variable: Food neophobia (CFNS)	-0,30	0,84	-0,39	0,18	0,53	0,37	0,70

Non-clincal sample

N = 51

Zmienna	Sensory sensitivity (ASRS)	**Food fussiness (CEBQ)**	Enjoyment of food (CEBQ)	Concern about child weight (CFQ)	Pressure to eat (CFQ)	**Monitoring (CFQ)**	**Selective eating (EDY-Q)**
Food neophobia (CFNS)	0,17	-0,25	-0,14	-0,04	-0,12	-0,25	-0,27
